# Approximate weakly efficient solutions of set-valued vector equilibrium problems

**DOI:** 10.1186/s13660-018-1773-0

**Published:** 2018-07-20

**Authors:** Jian Chen, Yihong Xu, Ke Zhang

**Affiliations:** Department of Mathematics, Nanchang University, Nanchang, China

**Keywords:** 90C33, 90C46, 90C59, Set-valued vector equilibrium problem, Approximate weakly efficient solution, Near cone-subconvexlikeness, Optimality condition

## Abstract

In this paper, we introduce a new kind of approximate weakly efficient solutions to the set-valued vector equilibrium problems with constraints in locally convex Hausdorff topological vector spaces; then we discuss a relationship between the weakly efficient solutions and approximate weakly efficient solutions. Under the assumption of near cone-subconvexlikeness, by using the separation theorem for convex sets we establish Kuhn–Tucker-type and Lagrange-type optimality conditions for set-valued vector equilibrium problems, respectively.

## Introduction

Vector optimization problems, vector variational inequality problems, vector complementarity problems, and vector saddle point problems are particular cases of vector equilibrium problems. As an extensive mathematical model, the vector equilibrium problem is a hot topic in the fields of operations research and nonlinear analysis (see [1–8]). Gong [2–4] obtained optimality conditions for vector equilibrium problems with constraints under the assumption of cone-convexity, and by using a nonlinear scalarization function and Ioffe subdifferentiability he derived optimality conditions for weakly efficient solutions, Henig solutions, super efficient solutions, and globally efficient solutions to nonconvex vector equilibrium problems. Long et al. [5] obtained optimality conditions for Henig efficient solutions to vector equilibrium problems with functional constrains under the assumption of near cone-subconvexlikeness. Luu et al. [7, 8] established sufficient and necessary conditions for efficient solutions to vector equilibrium problems with equality and inequality constraints and obtained the Fritz John and Karush–Kuhn–Tucker necessary optimality conditions for locally efficient solutions to vector equilibrium problems with constraints and sufficient conditions under assumptions of appropriate convexities.

It is well known that models describe only simplified versions of real problems and numerical algorithms generate only approximate solutions. Hence it is interesting and meaningful to have a theoretical analysis of the notion of an approximate solution. For example, Loridan [9, 10] introduced the concept of *ϵ*-solutions in general vector optimization problems.

As far as we know, there are few papers dealing with approximate weakly efficient solutions to the set-valued vector equilibrium problems. Li et al. [11] introduced a new kind of approximate solution set of a vector approximate equilibrium problem; it is uncertain if *ϵ* tends to zero, whether or not the approximate solution set equals to the original solution set? It is a natural question how to define approximate weakly efficient solutions to the set-valued vector equilibrium problems and under what condition the set of approximate weakly efficient solutions equals to the set of weakly efficient solutions? This has great theoretical significance and applicable value in the research of optimality conditions for approximate weakly efficient solutions to the set-valued vector equilibrium problems.

On the other hand, convexity plays an important role in the study of vector equilibrium problems. In 2001, Yang et al. [12] introduced a new convexity, named near cone-subconvexlikeness, and proved that it is a generalization of cone-convexness and cone-subconvexlikeness. In 2005, Sach (see [13]) introduced another new convexity called ic-cone-convexness, Xu et al. [14] proved that near cone-subconvexlikeness is also a generalization of ic-coneconvexness. Up to now, near cone-subconvexlikeness is considered to be the most generalized convexity.

Motivated by works in [3, 12, 15], in this paper, we introduce a new kind of approximate weakly efficient solutions to the set-valued vector equilibrium problems and reveal the relationship between weakly efficient solutions and approximate weakly efficient solutions. We establish Kuhn-Tucker type and Lagrange-type optimality conditions for set-valued vector equilibrium problems under the assumption of the near cone-subconvexlikeness.

The organization of the paper is as follows. Some preliminary facts are given in Sect. 2 for our later use. Section 3 is devoted to the relationship between weakly efficient solutions and approximate weakly efficient solutions. In Sect. 4, we establish Kuhn–Tucker-type sufficient and necessary optimality conditions for approximate weakly efficient solutions to the set-valued vector equilibrium problems. In Sect. 5, we establish Lagrange-type sufficient and necessary optimality conditions for approximate weakly efficient solutions to the set-valued vector equilibrium problems. At the end of the paper, we draw some conclusions.

## Preliminaries

Let *X* be a real topological vector space, and let *Y* and *Z* be real locally convex Hausdorff topological vector spaces with topological dual spaces $Y^{*}$ and $Z^{*}$, respectively. Let $C\subset Y$ and $D\subset Z$ be pointed closed convex cones with $\operatorname{int}C\neq \emptyset $ and $\operatorname{int}D\neq \emptyset $. The dual cones $C^{*}$ of *C* and $D^{*}$ of *D* are defined as $C^{*}=\{\phi \in Y^{*}:\phi (c)\geq 0, \forall c\in C\}$ and $D^{*}=\{\psi \in Z^{*}:\psi (d)\geq 0,\forall d\in D\}$, respectively. Let $X_{0}$ be a nonempty convex subset in *X*, and let $G:X_{0}\rightarrow 2^{Z}$ and $\Phi:X_{0}\times X_{0}\rightarrow 2^{Y}$ be mappings.

We denote by $L(Z,Y)$ the set of all continuous linear operators from *Z* to *Y*. A subset $L^{+}(Z,Y)$ of $L(Z,Y)$ is defined as $L^{+}(Z,Y)=\{T\in L(Z,Y):T(D)\subset C\}$.

We denote the feasible set by

$$ A=\bigl\{ x\in X_{0}:G(x)\cap (-D)\neq \emptyset \bigr\} . $$

Consider the set-valued vector equilibrium problem with constraints (for short, Φ-SVEPC): find $x\in A$ such that

$$ \Phi (x,y)\cap (-P)=\emptyset,\quad \forall y\in A, $$

where $P\cup \{0\}$ is a convex cone in *Y*.

### Definition 2.1

A vector $\bar{x}\in A$ satisfying

$$ \Phi (\bar{x},y)\cap (-\operatorname{int}C)=\emptyset, \quad \forall y\in A, $$

is called a weakly efficient solution to the Φ-SVEPC. The set of all weakly efficient solutions to the Φ-SVEPC is denoted by $X_{W\min }(\Phi,A)$.

Let $F:X_{0}\rightarrow 2^{Y}$ be a set-valued map. We consider the following set-valued optimization problem:

$$\begin{aligned} &\mathrm{(SOP)}\quad \min F(x), \\ &\hphantom{\mathrm{(SOP)}\quad}\text{s.t. } x\in A=\bigl\{ x\in X_{0}:G(x)\cap (-D) \neq \emptyset \bigr\} . \end{aligned}$$

We assume that the feasible set $A\subset X_{0}$ of (SOP) is nonempty.

### Definition 2.2

A feasible solution *x̄* of (SOP) is said to be a weakly efficient solution of (SOP) if there exists $\bar{y}\in F(\bar{x})$ such that $(F(A)-\bar{y})\cap (-\operatorname{int}C)=\emptyset$. In this case, $(\bar{x},\bar{y})$ is said to be a weakly efficient pair to (SOP).

### Definition 2.3

Let $\epsilon \in C$. A feasible solution *x̄* of (SOP) is said to be an *ϵ*-weakly efficient solution of (SOP) if there exists $\bar{y}\in F(\bar{x})$ such that $(F(A)-\bar{y}+\epsilon)\cap ( -\operatorname{int}C)=\emptyset$. In this case, $(\bar{x},\bar{y})$ is said to be an *ϵ*-weakly efficient pair to (SOP).

Let $\bar{T}\in L^{+}(Z,Y)$. Consider the following unconstrained set-valued optimization problem induced by (SOP):

$$ (\mathrm{USOP})_{\bar{T}}\quad \min_{x\in X_{0}} L(x,\bar{T}), $$

where $L(x,\bar{T})=F(x)+\bar{T}(G(x))$, $(x,\bar{T})\in X_{0} \times L^{+}(Z,Y)$.

### Definition 2.4

A vector $\bar{x}\in X_{0}$ is said to be a weakly efficient solution of $(\mathrm{USOP})_{\bar{T}}$ if there exists $\bar{y} \in F(\bar{x})$ such that $(L(X_{0},\bar{T})-\bar{y})\cap ( -\operatorname{int}C)=\emptyset$, where $L(X_{0},\bar{T})=\bigcup_{x\in X_{0}}L(x,\bar{T})$. In this case, $(\bar{x},\bar{y})$ is said to be a weakly efficient pair to $(\mathrm{USOP})_{\bar{T}}$.

### Definition 2.5

Let $\epsilon \in C$. A vector $\bar{x}\in X_{0}$ is said to be an *ϵ*-weakly efficient solution of $(\mathrm{USOP})_{\bar{T}}$ if $\exists \bar{y}\in F(\bar{x})$ such that $(L(X_{0},\bar{T})-\bar{y}+ \epsilon)\cap (-\operatorname{int}C)=\emptyset$, where $L(X_{0},\bar{T})= \bigcup_{x\in X_{0}}L(x,\bar{T})$. In this case, $(\bar{x}, \bar{y})$ is said to be an *ϵ*-weakly efficient pair to $(\mathrm{USOP})_{\bar{T}}$.

Several definitions of generalized convexities have been introduced in the literature.

### Definition 2.6

The map $F:X_{0}\rightarrow 2^{Y}$ is said to be *C*-convex on $X_{0}$ if, for all $x_{1},x_{2}\in X_{0}$ and $\lambda \in [0,1]$, we have

$$ \lambda F(x_{1})+(1-\lambda)F(x_{2})\subset F\bigl(\lambda x_{1}+(1- \lambda)x_{2}\bigr)+C. $$

### Definition 2.7

([16])

The map $F:X_{0}\rightarrow 2^{Y}$ is said to be *C*-subconvexlike on $X_{0}$ iff there exists $\theta \in \operatorname{int}C$ such that, for all $x_{1},x_{2}\in X_{0}$, $\lambda \in [0,1]$, $y_{i} \in F(x_{i})$, $i=1,2$, and $\alpha >0$, there exists $x_{3}\in X_{0}$ such that

$$ \alpha \theta +\lambda y_{1}+(1-\lambda)y_{2}\in F(x_{3})+C. $$

### Definition 2.8

([17])

The map $F:X_{0}\rightarrow 2^{Y}$ is said to be generalized *C*-subconvexlike on $X_{0}$ iff there exists $\theta \in \operatorname{int}C$ such that, for all $x_{1},x_{2}\in X_{0}$, $\lambda \in [0,1]$, and $\alpha >0$, there exist $x_{3}\in X_{0}$ and $\rho >0$ such that

$$ \alpha \theta +\lambda F(x_{1})+(1-\lambda)F(x_{2})\subset \rho F(x _{3})+C. $$

### Definition 2.9

([12])

The map $F:X_{0}\rightarrow 2^{Y}$ is called nearly *C*-subconvexlike on $X_{0}$ iff clcone$(F(X_{0})+C)$ is convex.

If $\emptyset \neq S_{1}\subset Y$, $\emptyset \neq S_{2}\subset Y$, $\bar{y}\in Y$, and $\psi \in Y^{*}$, then

$$ \psi (S_{1})\geq \psi (S_{2}) \text{ stands for } \psi (s _{1})\geq \psi (s_{2}), \quad \forall s_{1}\in S_{1}, s_{2}\in S_{2}, $$

and

$$ \psi (S_{1})\geq \psi (\bar{y}) \text{ stands for } \psi (s _{1})\geq \psi (\bar{y}), \quad \forall s_{1}\in S_{1}. $$

## Approximate weakly efficient solutions

Firstly, we introduce approximate weakly efficient solutions to the set-valued vector equilibrium problems with constraints.

### Definition 3.1

Let $\epsilon \in C$. A vector $\bar{x}\in A$ satisfying

$$ \bigl(\Phi (\bar{x},y)+\epsilon\bigr)\cap (-\operatorname{int}C)=\emptyset, \quad \forall y\in A, $$

is called an *ϵ*-weakly efficient solution to the Φ-SVEPC. The set of all *ϵ*-weakly efficient solutions to the Φ-SVEPC is denoted by $\epsilon \text{-}X_{W\min }(\Phi,A)$.

Let $\Upsilon:X_{0}\times X_{0}\rightarrow 2^{Y}$ be a mapping. Consider the following unconstrained set-valued vector equilibrium problem (for short, ϒ-USVEP): find $x\in X_{0}$ such that

$$ \Upsilon (x,y)\cap (-P)=\emptyset, \quad \forall y\in X_{0}, $$

where $P\cup \{0\}$ is a convex cone in *Y*.

### Definition 3.2

Let $\epsilon \in C$. A vector $\bar{x}\in X_{0}$ satisfying

$$ \bigl(\Upsilon (\bar{x},y)+\epsilon\bigr)\cap (-\operatorname{int}C)=\emptyset, \quad \forall y\in X_{0}, $$

is called an *ϵ*-weakly efficient solution to the ϒ-USVEP. The set of all *ϵ*-weakly efficient solutions to the ϒ-USVEP is denoted by $\epsilon \text{-}X_{W\min }( \Upsilon,X_{0})$.

### Proposition 3.1

*For any* $\epsilon \in C$, *we have*

$$ X_{W\min }(\Phi,A)\subset \epsilon \text{-}X_{W\min }(\Phi,A). $$

### Proof

If $x\notin \epsilon \text{-}X_{W\min }(\Phi,A)$, then there exists $\bar{y}\in A$ such that

$$ \bigl(\Phi (x,\bar{y})+\epsilon\bigr)\cap (-\operatorname{int}C)\ne \emptyset. $$

Thus there exists $\bar{z}\in \Phi (x,\bar{y})$ such that

3.1 $$ \bar{z}+\epsilon \in -\operatorname{int}C. $$

Since *C* is a convex cone, from $\epsilon \in C$ and (3.1) we have

$$ \bar{z}\in -\operatorname{int}C-\epsilon \subset -\operatorname{int}C-C\subset -\operatorname{int}C. $$

Hence

$$ \Phi (x,\bar{y})\cap (-\operatorname{int}C)\neq \emptyset, $$

and thus $x\notin X_{W\min }(\Phi,A)$. Then we obtain

$$ X_{W\min }(\Phi,A)\subset \epsilon \text{-}X_{W\min }(\Phi,A). $$

 □

Next, we show that in the proposition the relationship may be strict when $\epsilon \in C\setminus {\{0\}}$.

### Example 3.1

Let $X=R^{1}$, $A=[0,2]$, $Y=R^{2}$, $C=R_{+}^{2}$, and $\epsilon =(x_{0},y_{0})\in C\setminus {\{0\}}$. Let $\Phi:A\times A \longrightarrow 2^{Y}$ be defined by $\Phi (x,y)=\{(p,q):q\geq p^{2}-x \}\cap ([-y,y]\times [0,+\infty))$, $\forall x,y\in A$. It is obvious that $X_{W\min }(\Phi,A)=\{0\}$; however, $\epsilon \text{-}X_{W \min }(\Phi,A)=[0,\delta ]$, where $\delta =\min \{\max \{x_{0}^{2},y _{0}\},2\}$.

### Proposition 3.2

*For any* $\epsilon_{1},\epsilon_{2}\in C$, *if* $\epsilon_{2}- \epsilon_{1}\in C$, *then*

$$ \epsilon_{1}\text{-}X_{W\min }(\Phi,A)\subset \epsilon_{2} \text{-}X _{W\min }(\Phi,A). $$

### Proof

If $x\notin \epsilon_{2}\text{-}X_{W\min }(\Phi,A)$, then there exists $y_{1}\in A$ such that

$$ \bigl(\Phi (x,y_{1})+\epsilon_{2}\bigr)\cap (-\operatorname{int}C)\neq \emptyset. $$

Thus there exists $z_{1}\in \Phi (x,y_{1})$ such that

3.2 $$ z_{1}+\epsilon_{2}\in -\operatorname{int}C. $$

Since *C* is a convex cone, from $\epsilon_{2}-\epsilon_{1}\in C$ and (3.2) we have that

$$ z_{1}+\epsilon_{1}=z_{1}+\epsilon_{2}-( \epsilon_{2}-\epsilon_{1}) \in -\operatorname{int}C-C= -\operatorname{int}C. $$

Hence

$$ \bigl(\Phi (x,y_{1})+\epsilon_{1}\bigr)\cap (-\operatorname{int}C)\neq \emptyset, $$

and thus $x\notin \epsilon_{1}\text{-}X_{W\min }(\Phi,A)$, so we obtain

$$ \epsilon_{1}\text{-}X_{W\min }(\Phi,A)\subset \epsilon_{2}\text{-}X _{W\min }(\Phi,A). $$

 □

In what follows, we discuss the relationship between the approximate weakly efficient solutions and weakly efficient solutions to the set-valued vector equilibrium problems with constraints.

### Proposition 3.3

*We have*:

$$ \bigcap_{\epsilon \in C\setminus {\{0\}}}\epsilon \text{-}X _{W\min }( \Phi,A)=X_{W\min }(\Phi,A). $$

### Proof

Firstly, we prove that

$$ X_{W\min }(\Phi,A)\subset \bigcap_{\epsilon \in C\setminus {\{0\}}} \epsilon \text{-}X_{W\min }( \Phi,A). $$

From Proposition 3.1 we can see that, for any $\epsilon \in C \setminus {\{0\}}$, we have

$$ X_{W\min }(\Phi,A)\subset \epsilon \text{-}X_{W\min }(\Phi,A). $$

Hence

$$ X_{W\min }(\Phi,A)\subset \bigcap_{\epsilon \in C\setminus {\{0\}}} \epsilon \text{-}X_{W\min }( \Phi,A). $$

Next, we prove that

$$ \bigcap_{\epsilon \in C\setminus {\{0\}}}\epsilon \text{-}X _{W\min }( \Phi,A)\subset X_{W\min }(\Phi,A). $$

Suppose $\bar{x}\notin X_{W\min }(\Phi,A)$. Then there exists $y_{0}\in A$ such that

3.3 $$ \Phi (\bar{x},y_{0})\cap (-\operatorname{int}C)\neq \emptyset, $$

and hence we can find $z_{0}\in \Phi (\bar{x},y_{0})$ such that

$$ z_{0}\in -\operatorname{int}C. $$

Then there exists a neighborhood $U_{0}$ of 0 in *Y* such that

$$ z_{0}+U_{0}\subset -\operatorname{int}C. $$

Choosing $\epsilon_{0}\in (C\cap U_{0})\setminus {\{0\}}$, we have

$$ z_{0}+\epsilon_{0}\in -\operatorname{int}C. $$

Since $z_{0}\in \Phi (\bar{x},y_{0})$, we have

$$ \bigl(\Phi (\bar{x},y_{0})+\epsilon_{0}\bigr)\cap (- \operatorname{int}C)\neq \emptyset. $$

Hence $\bar{x}\notin \epsilon_{0}\text{-}X_{W\min }(\Phi,A)$, and therefore

$$ \bar{x}\notin \bigcap_{\epsilon \in C\setminus {\{0\}}}\epsilon \text{-}X_{W\min }(\Phi,A). $$

Thus

$$ \bigcap_{\epsilon \in C\setminus {\{0\}}}\epsilon \text{-}X _{W\min }( \Phi,A)\subset X_{W\min }(\Phi,A). $$

From this we obtain

$$ \bigcap_{\epsilon \in C\setminus {\{0\}}}\epsilon \text{-}X _{W\min }( \Phi,A)=X_{W\min }(\Phi,A). $$

 □

## Kuhn–Tucker-type optimality conditions

In this section, under the assumption of near *C*-subconvexlikeness, we establish Kuhn–Tucker-type sufficient and necessary optimality conditions for approximate weakly efficient solutions to the set-valued vector equilibrium problems, which generalize the relevant results given by Gong [2] and Yang [17].

### Definition 4.1

Let $\bar{x}\in X_{0}$, and let $\varphi:X_{0}\rightarrow 2^{Y\times Z}$ be an ordered pair mapping defined as $\varphi (x)=(\Phi (\bar{x},x)+ \epsilon,G(x))$, $\forall x\in X_{0}$.

By definition, *φ* is nearly $C\times D$-subconvexlike on $X_{0}$ if and only if $\operatorname{cl}(\operatorname{cone}(\varphi (X_{0})+C\times D))$ is convex, where $\varphi (X_{0})=\bigcup_{x\in X_{0}}\varphi (x)= \bigcup_{x\in X_{0}}(\Phi (\bar{x},x)+\epsilon,G(x))$.

### Lemma 4.1

([15])

*If* $y^{*}\in C^{*}\setminus \{0_{Y^{*}}\}$, $c_{0}\in \operatorname{int}C$, *then* $y^{*}(c_{0})>0$.

### Theorem 4.1

*Suppose that* *φ* *is nearly* $C\times D$-*subconvexlike on* $X_{0}$ *and that there exists* $x_{0}\in X_{0}$ *such that* $G(x_{0}) \cap (-\operatorname{int}D)\neq \emptyset $. *If* *x̄* *is an* *ϵ*-*weakly efficient solution to the* Φ-*SVEPC*, *then there exist* $s^{*}\in C^{*}\setminus \{0_{Y^{*}}\}$ *and* $k^{*}\in D^{*}$ *such that*

$$ s^{*}(y)+s^{*}(\epsilon)+k^{*}(z)\geq 0, \quad \forall x\in X_{0},y\in \Phi (\bar{x},x),z\in G(x). $$

### Proof

Since *x̄* is an *ϵ*-weakly efficient solution to the Φ-SVEPC, we have

4.1 $$ \bigl(\Phi (\bar{x},x)+\epsilon\bigr)\cap (-\operatorname{int}C)=\emptyset, \quad \forall x\in A. $$

Next, we prove that

4.2 $$ \bigl(\operatorname{cone}\bigl(\varphi (X_{0})+C\times D\bigr)\bigr) \cap \bigl((-\operatorname{int}C) \times (-\operatorname{int}D)\bigr)=\emptyset. $$

Suppose to the contrary that there exist $\hat{\lambda }\geq 0$ and $\hat{x}\in X_{0}$ such that

$$ \bigl(\hat{\lambda }\bigl(\Phi (\bar{x},\hat{x})+C+\epsilon,G(\hat{x})+D\bigr) \bigr) \cap \bigl((-\operatorname{int}C)\times (-\operatorname{int}D)\bigr)\neq \emptyset. $$

Thus,

4.3 $$ \bigl(\hat{\lambda }\bigl(\Phi (\bar{x},\hat{x})+C+\epsilon\bigr) \bigr)\cap ( -\operatorname{int}C)\neq \emptyset $$

and

4.4 $$ \bigl(\hat{\lambda }\bigl(G(\hat{x})+D\bigr)\bigr)\cap (-\operatorname{int}D)\neq \emptyset. $$

From $0\notin -\operatorname{int}D$ we have $\hat{\lambda }>0$. Since *D* is a convex cone, combining with (4.4), we have

$$ G(\hat{x})\cap (-\operatorname{int}D)\neq \emptyset. $$

It is clear that

$$ G(\hat{x})\cap (-D)\neq \emptyset. $$

Thus $\hat{x}\in A$. Since $\operatorname{int}C\cup \{0\}$ is a cone, by (4.3) we get

$$ \bigl(\Phi (\bar{x},\hat{x})+C+\epsilon\bigr)\cap (-\operatorname{int}C)\neq \emptyset. $$

Since *C* is a convex cone, we have

$$ \bigl(\Phi (\bar{x},\hat{x})+\epsilon\bigr)\cap (-\operatorname{int}C)\neq \emptyset, $$

which contradicts (4.1), and thus we obtain (4.2).

Since $-\operatorname{int}C$ and $-\operatorname{int}D$ are open sets, combining with (4.2), we have

4.5 $$ \bigl(\operatorname{cl}\bigl(\operatorname{cone}\bigl(\varphi (X_{0})+C\times D \bigr)\bigr)\bigr)\cap \bigl((-\operatorname{int}C) \times (-\operatorname{int}D)\bigr)=\emptyset. $$

Since *φ* is nearly $C\times D$-subconvexlike on $X_{0}$, by Definition 4.1 we can see that $\operatorname{cl}(\operatorname{cone}(\varphi (X_{0})+C \times D))$ is convex. By the separation theorem for convex sets, there exists $(s^{*},k^{*})\in Y^{*}\times Z^{*}\setminus \{(0_{Y^{*}},0_{Z ^{*}})\}$ such that

$$ \bigl(s^{*},k^{*}\bigr) \bigl(\operatorname{cl}\bigl(\operatorname{cone}\bigl(\varphi (X_{0})+C\times D\bigr)\bigr)\bigr)\geq s^{*}( -\operatorname{int}C)+k^{*}(-\operatorname{int}D). $$

Since $\operatorname{cl}(\operatorname{cone}(\varphi (X_{0})+C\times D))$ is a cone, and there is a lower bound of $(s^{*},k^{*})$ on $\operatorname{cl}(\operatorname{cone}(\varphi (X_{0})+C\times D))$, we have

$$ \bigl(s^{*},k^{*}\bigr) \bigl(\operatorname{cl}\bigl(\operatorname{cone}\bigl(\varphi (X_{0})+C\times D\bigr)\bigr)\bigr)\geq 0. $$

Hence

4.6 $$ \bigl(s^{*},k^{*}\bigr) \bigl(\varphi (X_{0})+C\times D\bigr)\geq 0. $$

Since $(0_{Y},0_{Z})\in C\times D$, we have

$$ \bigl(s^{*},k^{*}\bigr) \bigl(\varphi (X_{0}) \bigr)\geq 0. $$

Thus

$$ s^{*}\bigl(\Phi (\bar{x},x)+\epsilon\bigr)+k^{*}\bigl(G(x) \bigr)\geq 0, \quad \forall x\in X _{0}. $$

It is clear that

4.7 $$ s^{*}(y)+s^{*}(\epsilon)+k^{*}(z) \geq 0, \quad \forall x\in X_{0},y\in \Phi (\bar{x},x),z\in G(x). $$

From (4.6) we get

4.8 $$\begin{aligned}& s^{*}(y+\epsilon +\delta_{1}c)+k^{*}(z+ \delta_{2}d) \\& \quad \geq 0, \quad \forall x\in X_{0}, y\in \Phi (\bar{x},x),z\in G(x),\delta_{1},\delta_{2} \geq 0,c\in C,d\in D. \end{aligned}$$

Next, we prove that

$$ s^{*}(c)\geq 0, \quad \forall c\in C. $$

Suppose to the contrary that there exists $c_{0}\in C$ such that

$$ s^{*}(c_{0})< 0. $$

When $\delta_{1}$ is large enough, there exist $x_{1}\in X_{0},y_{1} \in \Phi (\bar{x},x_{1}),z_{1}\in G(x_{1}),\delta_{2}^{\prime }\geq 0$, and $d_{1}\in D$ such that

$$ \delta_{1}s^{*}(c_{0})< -s^{*}(y_{1}+ \epsilon)-k^{*}\bigl(z_{1}+\delta_{2} ^{\prime }d_{1}\bigr), $$

which contradicts (4.8). Hence we obtain

$$ s^{*}(c)\geq 0, \quad \forall c\in C. $$

Similarly, we get

$$ k^{*}(d)\geq 0, \quad \forall d\in D. $$

Thus

$$ s^{*}\in C^{*},\qquad k^{*}\in D^{*}. $$

Then we need to prove that

$$ s^{*}\neq 0_{Y^{*}}. $$

Suppose to the contrary that

$$ s^{*}=0_{Y^{*}}. $$

Since $(s^{*},k^{*})\neq (0_{Y^{*}},0_{Z^{*}})$, we have

$$ k^{*}\neq 0_{Z^{*}}. $$

From $k^{*}\in D^{*}\setminus \{0_{Z^{*}}\}$, $s^{*}=0_{Y^{*}}$, and (4.7) we can see that

4.9 $$ k^{*}\bigl(G(x)\bigr)\geq 0, \quad \forall x\in X_{0}. $$

On the other hand, there exists $x_{0}\in X_{0}$ such that $G(x_{0}) \cap (-\operatorname{int}D)\neq \emptyset $, and thus there exists $p\in G(x_{0})\cap (-\operatorname{int}D)$, so that by Lemma 4.1 we obtain

$$ k^{*}(p)< 0, $$

which contradicts (4.9). Hence

$$ s^{*}\in C^{*}\setminus \{0_{Y^{*}}\}. $$

The proof is complete. □

### Corollary 4.1

*Suppose that* $\bar{x}\in A$, $0\in \Phi (\bar{x},\bar{x})$, *φ* *is nearly* $C\times D$-*subconvexlike on* $X_{0}$, *and there exists* $x_{0}\in X_{0}$ *such that* $G(x_{0})\cap (-\operatorname{int}D) \neq \emptyset $. *If* *x̄* *is a weakly efficient solution to the* Φ-*SVEPC*, *then there exist* $s^{*}\in C^{*}\setminus \{0_{Y^{*}} \}$ *and* $k^{*}\in D^{*}$ *such that* $\min k^{*}(G(\bar{x}))=0$ *and*

4.10 $$ \min \bigl\{ s^{*}(y)+k^{*}(z):x\in X_{0},y\in \Phi (\bar{x},x),z\in G(x)\bigr\} =0. $$

### Proof

In the proof of Theorem 4.1, letting $\epsilon =0$, we see that

4.11 $$ s^{*}(y)+k^{*}(z)\geq 0, \quad \forall x\in X_{0}, y\in \Phi (\bar{x},x), z\in G(x). $$

From $\bar{x}\in A$ we have

$$ G(\bar{x})\cap (-D)\neq \emptyset. $$

Thus there exists $q\in G(\bar{x})$ such that $q\in -D$, and since $k^{*}\in D^{*}$, we have

4.12 $$ k^{*}(q)\leq 0. $$

Letting $x=\bar{x}$ in (4.11), by $0\in \Phi (\bar{x},\bar{x})$ we have

4.13 $$ k^{*}(z)\geq 0, \quad \forall z\in G(\bar{x}). $$

From $q\in G(\bar{x})$ we have $k^{*}(q)\geq 0$. Combining with (4.12), we obtain

$$ k^{*}(q)=0. $$

Thus

4.14 $$ 0\in k^{*}\bigl(G(\bar{x})\bigr). $$

Combining with (4.13), we get

$$ \min k^{*}\bigl(G(\bar{x})\bigr)=0. $$

From $0\in \Phi (\bar{x},\bar{x})$ and (4.14) it follows that

$$ 0\in s^{*}\bigl(\Phi (\bar{x},\bar{x})\bigr)+k^{*}\bigl(G( \bar{x})\bigr). $$

Combining with (4.11), we obtain (4.10). The proof is complete. □

### Theorem 4.2

*Assume that*

(i) $\bar{x}\in A$ *and*
(ii) *there exist* $s^{*}\in C^{*}\setminus \{0_{Y ^{*}}\}$ *and* $k^{*}\in D^{*}$ *such that*
  $$ s^{*}(y)+s^{*}(\epsilon)+k^{*}(z)\geq 0, \quad \forall x\in X_{0},y\in \Phi (\bar{x},x),z\in G(x). $$

*Then* *x̄* *is an* *ϵ*-*weakly efficient solution to the* Φ-*SVEPC*.

### Proof

Suppose to the contrary that *x̄* is not an *ϵ*-weakly efficient solution to the Φ-SVEPC. Then we can find $\hat{x} \in A$ such that

$$ \bigl(\Phi (\bar{x},\hat{x})+\epsilon\bigr)\cap (-\operatorname{int}C)\neq \emptyset. $$

Thus there exists $\hat{y}\in \Phi (\bar{x},\hat{x})$ such that

$$ \hat{y}+\epsilon \in -\operatorname{int}C. $$

By $s^{*}\in C^{*}\setminus \{0_{Y^{*}}\}$ and Lemma 4.1 we have

4.15 $$ s^{*}(\hat{y})+s^{*}(\epsilon)< 0. $$

Choosing $\hat{z}\in G(\hat{x})\cap (-D)$, since $k^{*}\in D^{*}$, we have $k^{*}(\hat{z})\leq 0$. Combining with (4.15), we obtain

$$ s^{*}(\hat{y})+s^{*}(\epsilon)+k^{*}(\hat{z})< 0, $$

which contradicts (ii), and hence *x̄* is an *ϵ*-weakly efficient solution to the Φ-SVEPC. □

### Corollary 4.2

*Assume that*

(i) $\bar{x}\in A$ *and*
(ii) *there exist* $s^{*}\in C^{*}\setminus \{0_{Y ^{*}}\}$ *and* $k^{*}\in D^{*}$ *such that*
  $$ s^{*}(y)+k^{*}(z)\geq 0, \quad \forall x\in X_{0},y\in \Phi (\bar{x},x),z \in G(x). $$

*Then* *x̄* *is a weakly efficient solution to the* Φ-*SVEPC*.

### Proof

Letting $\epsilon =0$ in Theorem 4.2, we get the conclusion. □

### Corollary 4.3

*Suppose that* $\bar{x}\in A$, $0\in \Phi (\bar{x},\bar{x})$, *φ* *is nearly* $C\times D$-*subconvexlike on* $X_{0}$, *and there exists* $x_{0}\in X_{0}$ *such that* $G(x_{0})\cap (-\operatorname{int}D) \neq \emptyset $. *Then* *x̄* *is a weakly efficient solution to the* Φ-*SVEPC if and only if there exist* $s^{*}\in C^{*}\setminus \{0_{Y ^{*}}\}$ *and* $k^{*}\in D^{*}$ *such that* $\min k^{*}(G(\bar{x}))=0$ *and*

$$ \min \bigl\{ s^{*}(y)+k^{*}(z):x\in X_{0},y\in \Phi (\bar{x},x),z\in G(x)\bigr\} =0. $$

### Proof

This follows directly from Corollaries 4.1 and 4.2. □

### Remark 4.1

Corollary 4.3 extends Theorem 3.1 of Gong [2] in the following aspects:

(i) The vector-valued function is extended to a set-valued function;
(ii) The cone-convexity of *φ* is extended to near cone-subconvexlikeness.

### Corollary 4.4

*Suppose that* $\bar{x}\in X_{0}$, $\bar{y}\in F(\bar{x})$, $(F-\bar{y},G)$ *is nearly* $C\times D$-*subconvexlike on* $X_{0}$, *and there exists* $x_{0}\in X_{0}$ *such that* $G(x_{0})\cap (-\operatorname{int}D) \neq \emptyset $. *If* $(\bar{x},\bar{y})$ *is a weakly efficient pair to the* (*SOP*), *then there exist* $s^{*}\in C^{*}\setminus \{0_{Y^{*}}\}$ *and* $k^{*}\in D^{*}$ *such that* $\min k^{*}(G(\bar{x}))=0$ *and*

$$ s^{*}(\bar{y})=\min \bigl\{ s^{*}\bigl(F(x) \bigr)+k^{*}\bigl(G(x)\bigr):x\in X_{0}\bigr\} . $$

### Proof

Letting $\Phi (y,x)=F(x)-\bar{y}$, it is clear that $\Phi (y,x)$ depends only upon the second variable. Since $\bar{y}\in F(\bar{x})$, we have $0\in F(\bar{x})-\bar{y}$, and hence $0\in \Phi (\bar{x},\bar{x})$ and

$$ \bigl(F(x)-\bar{y},G(x)\bigr)=\bigl(\Phi (\bar{x},x),G(x)\bigr). $$

Since $(\bar{x},\bar{y})$ is a weakly efficient pair to the (SOP), we can see that *x̄* is a weakly efficient solution to the Φ-SVEPC. By Corollary 4.1 we get the conclusion. □

### Remark 4.2

From Remarks 3.1 and 3.3 in [18] we can see that if $(F-\bar{y},G)$ is generalized $C\times D$-subconvexlike on $X_{0}$, then $(F-\bar{y},G)$ is nearly $C\times D$-subconvexlike on $X_{0}$. Thus, Corollary 4.4 generalizes Theorem 4.2 in [17].

From Theorems 4.1 and 4.2 we obtain the following result.

### Corollary 4.5

*Suppose that* $\bar{x}\in A$, *φ* *is nearly* $C\times D$-*subconvexlike on* $X_{0}$ *and that there exists* $x_{0} \in X_{0}$ *such that* $G(x_{0})\cap (-\operatorname{int}D)\neq \emptyset $. *Then* *x̄* *is an* *ϵ*-*weakly efficient solution to the* Φ-*SVEPC if and only if there exist* $s^{*}\in C^{*}\setminus \{0_{Y ^{*}}\}$ *and* $k^{*}\in D^{*}$ *such that*

$$ s^{*}(y)+s^{*}(\epsilon)+k^{*}(z)\geq 0, \quad \forall x\in X_{0},y\in \Phi (\bar{x},x),z\in G(x). $$

## Lagrange-type optimality conditions

In this section, we present Lagrange-type sufficient and necessary optimality conditions for approximate weakly efficient solutions to the set-valued vector equilibrium problems, which generalize the relevant results given by Rong [15].

### Theorem 5.1

*Suppose that* $\bar{x}\in A$, $0\in \Phi (\bar{x},\bar{x})$, *φ* *is nearly* $C\times D$-*subconvexlike on* $X_{0}$, *and there exists* $x_{0}\in X_{0}$ *such that* $G(x_{0})\cap (-\operatorname{int}D) \neq \emptyset $. *If* *x̄* *is an* *ϵ*-*weakly efficient solution to the* Φ-*SVEPC*, *then there exists* $\bar{T}\in L^{+}(Z,Y)$ *such that* $-\bar{T}(G(\bar{x})\cap (-D))\subset ((\operatorname{int}C\cap \{0\})\setminus (\epsilon +\operatorname{int}C))$, *and* *x̄* *is an* *ϵ*-*weakly efficient solution to the* Ψ-*USVEP*, *where* $\Psi:X_{0}\times X_{0}\rightarrow 2^{Y}$ *is defined by*

$$ \Psi (y,x)=\Phi (y,x)+\bar{T}\bigl(G(x)\bigr). $$

### Proof

From the proof of Theorem 4.1 we see that there exist $s^{*}\in C^{*}\setminus \{0_{Y^{*}}\}$ and $k^{*}\in D^{*}$ satisfying (4.7).

Since $s^{*}\in C^{*}\setminus \{0_{Y^{*}}\}$, we can find $c_{0} \in \operatorname{int}C$ such that $s^{*}(c_{0})=1$. Define the operator $\bar{T}:Z\rightarrow Y$ by

$$ \bar{T}(z)=k^{*}(z)c_{0}, \quad \forall z\in Z. $$

Thus $\bar{T}(D)=k^{*}(D)c_{0}\subset C$. It is evident that

$$ \bar{T}\in L^{+}(Z,Y). $$

Letting $x=\bar{x}$ in (4.7), since $0\in \Phi (\bar{x},\bar{x})$, we have

5.1 $$ s^{*}(\epsilon)+k^{*}(z)\geq 0, \quad \forall z\in G(\bar{x})\cap (-D). $$

Noticing that $z\in -D$, we obtain

$$ -\bar{T}(z)=-k^{*}(z)c_{0}\in \operatorname{int}C\cup \{0\}. $$

Thus

5.2 $$ -\bar{T}\bigl(G(\bar{x})\cap (-D)\bigr)\subset \operatorname{int}C\cup \{0 \}. $$

Next, we prove

5.3 $$ -\bar{T}\bigl(G(\bar{x})\cap (-D)\bigr)\cap (\epsilon +\operatorname{int}C)=\emptyset. $$

Suppose to the contrary that there exists $\tilde{z}\in G(\bar{x}) \cap (-D)$ such that

5.4 $$ -\bar{T}(\tilde{z})\in \epsilon +\operatorname{int}C. $$

Thus $-\bar{T}(\tilde{z})-\epsilon \in \operatorname{int}C$. By the definition of *T̄* we have

$$ s^{*}\bigl(-\bar{T}(\tilde{z})-\epsilon\bigr)=s^{*} \bigl(-k^{*}(\tilde{z})c_{0}- \epsilon\bigr)= - \bigl(s^{*}(\epsilon)+k^{*}(\tilde{z})\bigr). $$

Combining with (5.1), we have

5.5 $$ s^{*}\bigl(-\bar{T}(\tilde{z})-\epsilon\bigr)\leq 0. $$

On the other hand, from $s^{*}\in C^{*}\setminus \{0_{Y^{*}}\}$ and Lemma 4.1 it follows that

$$ s^{*}(\operatorname{int}C)>0, $$

which, together with (5.5), gives

$$ -\bar{T}(\tilde{z})-\epsilon \notin \operatorname{int}C, $$

which contradicts (5.4). Thus we obtain (5.3).

The combination of (5.2) and (5.3) leads to

$$ -\bar{T}\bigl(G(\bar{x})\cap (-D)\bigr)\subset \bigl(\bigl(\operatorname{int}C\cap \{0\} \bigr)\setminus (\epsilon +\operatorname{int}C)\bigr). $$

Finally, we prove that *x̄* is an *ϵ*-weakly efficient solution to the Ψ-USVEP.

In fact, by the definition of *T̄* and (4.7) we obtain

$$ s^{*}\bigl(\Phi (\bar{x},x)+\bar{T}\bigl(G(x)\bigr)+\epsilon \bigr)=s^{*}\bigl(\Phi (\bar{x},x)\bigr) +s^{*}( \epsilon)+k^{*}\bigl(G(x)\bigr)\geq 0, \quad \forall x\in X_{0}. $$

Since $s^{*}(-\operatorname{int}C)<0$, we have

$$ \bigl(\Phi (\bar{x},x)+\bar{T}\bigl(G(x)\bigr)+\epsilon\bigr)\cap (-\operatorname{int}C)= \emptyset, \quad \forall x\in X_{0}. $$

Consequently,

$$ \bigl(\Psi (\bar{x},x)+\epsilon\bigr)\cap (-\operatorname{int}C)=\emptyset, \quad \forall x\in X_{0}. $$

It is evident that *x̄* is an *ϵ*-weakly efficient solution to the Ψ-USVEP. □

### Theorem 5.2

*Assume that*

(i) $\bar{x}\in A$ *and*
(ii) *there exists* $\bar{T}\in L^{+}(Z,Y)$ *such that* *x̄* *is an* *ϵ*-*weakly efficient solution to the* Ψ-*USVEP*, *where* $\Psi:X_{0}\times X_{0}\rightarrow 2^{Y}$ *is defined by*
  $$ \Psi (y,x)=\Phi (y,x)+\bar{T}\bigl(G(x)\bigr). $$

*Then* *x̄* *is an* *ϵ*-*weakly efficient solution to the* Φ-*SVEPC*.

### Proof

Since *x̄* is an *ϵ*-weakly efficient solution to the Ψ-USVEP, we have

$$ \biggl(\bigcup_{x\in X_{0}}\bigl(\Psi (\bar{x},x)+\epsilon \bigr)\biggr)\cap ( -\operatorname{int}C)=\emptyset. $$

Since *C* is a convex cone, we obtain

5.6 $$ \biggl(\bigcup_{x\in X_{0}}\bigl(\Psi ( \bar{x},x)+C+\epsilon\bigr)\biggr)\cap ( -\operatorname{int}C)=\emptyset. $$

On the other hand, for any $x\in A$, we have $G(x)\cap (-D)\neq \emptyset $. Thus there exists $z_{x}\in G(x)$ such that $z_{x}\in -D$. It follows from $\bar{T}\in L^{+}(Z,Y)$ that $\bar{T}(z_{x})\in -C$, thus $C-\bar{T}(z_{x})\subset C+C\subset C$, and hence $C\subset \bar{T}(z_{x})+C$; since $z_{x}\in G(x)$, it is evident that $C\subset \bar{T}(G(x))+C$.

Thus

$$\begin{aligned} \bigcup_{x\in A}\bigl(\Phi (\bar{x},x)+C+\epsilon\bigr) &\subset \bigcup_{x\in A}\bigl(\Phi (\bar{x},x)+\bar{T} \bigl(G(x)\bigr)+C+\epsilon\bigr) \\ &\subset \bigcup_{x\in X_{0}}\bigl(\Phi (\bar{x},x)+\bar{T} \bigl(G(x)\bigr)+C +\epsilon\bigr) \\ &=\bigcup_{x\in X_{0}}\bigl(\Psi (\bar{x},x)+C+\epsilon \bigr). \end{aligned}$$

It follows from (5.6) that

$$ \biggl(\bigcup_{x\in A}\bigl(\Phi (\bar{x},x)+C+ \epsilon\bigr)\biggr)\cap ( -\operatorname{int}C)= \emptyset. $$

Since $0\in C$, it is evident that

$$ \biggl(\bigcup_{x\in A}\bigl(\Phi (\bar{x},x)+\epsilon \bigr)\biggr)\cap ( -\operatorname{int}C)= \emptyset. $$

Hence *x̄* is an *ϵ*-weakly efficient solution to the Φ-SVEPC. □

From Theorems 5.1 and 5.2 we obtain the following result.

### Corollary 5.1

*Suppose that* $\bar{x}\in A$, $0\in \Phi (\bar{x},\bar{x})$, *φ* *is nearly* $C\times D$-*subconvexlike on* $X_{0}$, *and there exists* $x_{0}\in X_{0}$ *such that* $G(x_{0})\cap (-\operatorname{int}D) \neq \emptyset $. *Then* *x̄* *is an* *ϵ*-*weakly efficient solution to the* Φ-*SVEPC if and only if there exists* $\bar{T} \in L^{+}(Z,Y)$ *such that* *x̄* *is an* *ϵ*-*weakly efficient solution to the* Ψ-*USVEP*, *where* $\Psi:X_{0}\times X_{0}\rightarrow 2^{Y}$ *is defined by*

$$ \Psi (y,x)=\Phi (y,x)+\bar{T}\bigl(G(x)\bigr). $$

### Corollary 5.2

*Suppose that* $(F-\bar{y},G)$ *is nearly* $C\times D$-*subconvexlike on* $X_{0}$ *and there exists* $x_{0}\in X_{0}$ *such that* $G(x_{0})\cap ( -\operatorname{int}D)\neq \emptyset $. *If* $(\bar{x},\bar{y})$ *is an* *ϵ*-*weakly efficient pair to the* (*SOP*), *then there exists* $\bar{T}\in L^{+}(Z,Y)$ *such that* $-\bar{T}(G(\bar{x})\cap (-D)) \subset ((\operatorname{int}C\cap \{0\})\setminus (\epsilon +\operatorname{int}C))$, *and* $(\bar{x},\bar{y})$ *is an* *ϵ*-*weakly efficient pair to the* $(\mathrm{USOP})_{\bar{T}}$.

### Proof

Letting $\Phi (y,x)=F(x)-\bar{y}$, since $\bar{y}\in F(\bar{x})$, it is evident that $0\in \Phi (\bar{x},\bar{x})$ and $(F(x)-\bar{y},G(x))=( \Phi (\bar{x},x),G(x))$.

Since $(\bar{x},\bar{y})$ is an *ϵ*-weakly efficient pair to the (SOP), we see that *x̄* is an *ϵ*-weakly efficient solution to the Φ-SVEPC.

Thus by Theorem 5.1 there exists $\bar{T}\in L^{+}(Z,Y)$ such that $-\bar{T}(G(\bar{x})\cap (-D))\subset ((\operatorname{int}C\cap \{0\}) \setminus (\epsilon +\operatorname{int}C))$ and *x̄* is an *ϵ*-weakly efficient solution to the Ψ-USVEP, that is,

$$ \biggl(\bigcup_{x\in X_{0}}\bigl(\Phi (\bar{x},x)+\bar{T} \bigl(G(x)\bigr) +\epsilon\bigr)\biggr)\cap (-\operatorname{int}C)=\emptyset. $$

Consequently,

$$ \biggl(\bigcup_{x\in X_{0}}\bigl(F(x)-\bar{y}+\bar{T} \bigl(G(x)\bigr) +\epsilon\bigr)\biggr) \cap (-\operatorname{int}C)=\emptyset, $$

which is equivalent to

$$ \biggl(\bigcup_{x\in X_{0}}L(x,\bar{T})+\epsilon -\bar{y} \biggr)\cap ( -\operatorname{int}C)=\emptyset. $$

Thus

$$ \bigl(L(X_{0},\bar{T})+\epsilon -\bar{y}\bigr)\cap (-\operatorname{int}C)= \emptyset. $$

Hence, by Definition 2.5, $(\bar{x},\bar{y})$ is an *ϵ*-weakly efficient pair to the $(\mathrm{USOP})_{\bar{T}}$. □

### Remark 5.1

If $(F,G)$ is $C\times D$-subconvexlike, then $(F-\bar{y},G)$ is nearly $C\times D$-subconvexlike. Thus Corollary 5.2 generalizes Theorem 3.1 in [15].

### Corollary 5.3

*Assume that*

(i) $\bar{x}\in A, \bar{y}\in F(\bar{x})$, *and*
(ii) *there exists* $\bar{T}\in L^{+}(Z,Y)$ *such that* $(\bar{x},\bar{y})$ *is an* *ϵ*-*weakly efficient pair to the* $(\mathrm{USOP}) _{\bar{T}}$.

*Then* $(\bar{x},\bar{y})$ *is an* *ϵ*-*weakly efficient pair to the* (*SOP*).

### Proof

Letting $\Phi (y,x)=F(x)-\bar{y}$, since $\bar{y}\in F(\bar{x})$, it is evident that $0\in \Phi (\bar{x},\bar{x})$ and $(F(x)-\bar{y},G(x))=( \Phi (\bar{x},x),G(x))$.

Since $(\bar{x},\bar{y})$ is an *ϵ*-weakly efficient pair to the $(\mathrm{USOP})_{\bar{T}}$, we see that *x̄* is an *ϵ*-weakly efficient solution to the Ψ-USVEP. Combining this with Theorem 5.2, we conclude that *x̄* is an *ϵ*-weakly efficient solution to the Φ-SVEPC; it is clear that $(\bar{x}, \bar{y})$ is an *ϵ*-weakly efficient pair to the (SOP). □

### Remark 5.2

Comparing with Theorem 3.2 in [15], this corollary is not required for the convexity of $(F, G)$.

### Corollary 5.4

*Suppose that* $\bar{x}\in A$, $\bar{y}\in F(\bar{x})$, $(F-\bar{y},G)$ *is nearly* $C\times D$-*subconvexlike on* $X_{0}$, *and there exists* $x_{0}\in X_{0}$ *such that* $G(x_{0})\cap (-\operatorname{int}D)\neq \emptyset $. *Then* $(\bar{x},\bar{y})$ *is an* *ϵ*-*weakly efficient pair to the* (*SOP*) *if and only if there exists* $\bar{T}\in L^{+}(Z,Y)$ *such that* $(\bar{x},\bar{y})$ *is an* *ϵ*-*weakly efficient pair to the* $(\mathrm{USOP}) _{\bar{T}}$.

### Proof

This follows directly from Corollaries 5.2 and 5.3. □

## Conclusions

In this paper, we discuss some relationships between approximate weakly efficient solutions and weakly efficient solutions of set-valued vector equilibrium problems. We conclude that $\bigcap_{\epsilon \in C\setminus {\{0\}}}\epsilon \text{-}X_{W \min }(\Phi,A)=X_{W\min }(\Phi,A)$, and hence it is really “approximate”. The optimality conditions for set-valued vector equilibrium problems are established, and the results we obtained generalize those of Gong[2], Yang[17], and Rong[15]. As an extensive mathematical model, further research on approximate weakly efficient solutions of set-valued vector equilibrium problems seems to be of interest and value.
